# The acoustic adaptation hypothesis in a widely distributed South American frog: Southernmost signals propagate better

**DOI:** 10.1038/s41598-018-25359-y

**Published:** 2018-05-03

**Authors:** Nelson A. Velásquez, Felipe N. Moreno-Gómez, Enzo Brunetti, Mario Penna

**Affiliations:** 1 Laboratorio de Communicación Animal, Facultad de Ciencias Básicas, Universidad Católica del Maule, San Miguel 3605, 3480112 Talca, Chile; 2 Laboratorio de Bioacústica y Ecología del Comportamiento Animal, Facultad de Ciencias Básicas, Universidad Católica del Maule, San Miguel 3605, 3480112 Talca, Chile; 3 Programa de Fisiología y Biofísica, ICBM, Facultad de Medicina, Universidad de Chile, Independencia, 1027 Santiago, Chile

## Abstract

Animal communication occurs in environments that affect the properties of signals as they propagate from senders to receivers. We studied the geographic variation of the advertisement calls of male *Pleurodema thaul* individuals from eight localities in Chile. Furthermore, by means of signal propagation experiments, we tested the hypothesis that local calls are better transmitted and less degraded than foreign calls (i.e. acoustic adaptation hypothesis). Overall, the advertisement calls varied greatly along the distribution of *P. thaul* in Chile, and it was possible to discriminate localities grouped into northern, central and southern stocks. Propagation distance affected signal amplitude and spectral degradation in all localities, but temporal degradation was only affected by propagation distance in one out of seven localities. Call origin affected signal amplitude in five out of seven localities and affected spectral and temporal degradation in six out of seven localities. In addition, in northern localities, local calls degraded more than foreign calls, and in southern localities the opposite was observed. The lack of a strict optimal relationship between signal characteristics and environment indicates partial concordance with the acoustic adaptation hypothesis. Inter-population differences in selectivity for call patterns may compensate for such environmental constraints on acoustic communication.

## Introduction

Animal communication involves senders that produce signals to which receivers respond with behavioral changes^1^. The environments in which animals communicate affect propagating acoustic signals so that the structure of the sounds reaching the receivers are altered versions of the original patterns produced by emitters^2^. Acoustic signals transmitted across long distances can experience attenuation and degradation^3^. Attenuation refers to decreases in signal amplitude as a function of the distance from the sound source^4^. In ideal conditions, sounds propagating in the air experience a 6-dB reduction in amplitude every time distance is doubled (i.e. “spherical spreading”)^5^. However, in natural environments additional attenuation (i.e. excess attenuation) occurs due to reflection, absorption, reverberation, and scattering^2^. These processes also affect the spectral structure of acoustic signals, generally by reducing the relative amplitude of high-frequency components or by narrowing the frequency range of the signals. The temporal structure of the transmitted sounds is also altered, mainly compromising amplitude modulation traits^6^.

The acoustic adaptation hypothesis states that, in order to overcome environmental constraints, signals are endowed with optimal characteristics for transmission in the senders’ native environment^7^. This hypothesis implies that signals used in long range communication experience less attenuation and degredation in native than in foreign habitats depending on differences in vegetation coverage^8,9^. Experiments using transmissions of pure tones^10,11^ and local or foreign vocalizations^12–15^ have been used to explore this hypothesis. Additionally, a number of studies, especially in birds and mammals, have shown that environmental conditions affect call characteristics^12,13,16–23^. However, other studies of vertebrates have failed to show such optimal relationship between environments and call characteristics^9,24–26^. Particularly in anurans, few studies have shown that environmental conditions influence call properties^14,27^, rather most studies have shown a lack of optimal relationships between habitat properties and call characteristics^9,11,15,28–31^. Furthermore, studies of the acoustic adaptation hypothesis have mostly focused on interspecific comparisons, which implies that factors such as body size and phylogenetic relationships could confound conclusions^9,32,33^. One of few instances where an intraspecific comparison was made has shown that the call degradation of two subspecies of the frog *Acris crepitans* (i.e. *A.c. crepitans* and *A.c. blanchardi*) is lower in grassland (i.e. open habitat, native of *A.c. blanchardi*) than in pine forest (i.e. closed habitat, native of *A.c. crepitans*)^14,34^.

Due to its extensive latitudinal distribution in Chile, the four-eyed frog, *Pleurodema thaul* (Anura: Leptodactilydae) is an opportune model for studies of the geographic variation of behavioral traits^35–38^. The geographic distribution of *P. thaul* spans from the Atacama Desert (27°06′S, 69°53′W^39^) to the Patagonian region (45°24′S, 72°42′W^40^). This range includes various environments, from northern desert landscapes where rainfall is scarce and daily temperature oscillates greatly to southern latitudes characterized by high precipitation and narrow thermal ranges^41^. Studies have shown that advertisement calls and genetic distance both vary among localities along the distribution range, and three bioacoustic groups are recognized in this species: northern, central, and southern. These groups differ mainly in the dominant frequency and depth of intra-pulse modulations of the advertisement calls that males emit^35^. In addition, males from the northern and central bioacoustic groups differ from the southern group in their selectivity to local or foreign calls^36^. In contrast with this behavioral selectivity, females from these bioacoustic groups do not differ in their phonotactic responses to local or foreign calls^38^.

Given that males call on the water surface of slow flowing creeks and pools over which acoustic signals propagate efficiently^11^ and thresholds for males’ evoked vocal responses are fairly sensitive, reaching about levels of auditory thresholds^42^, *P. thaul* individuals are capable of communicating over long distances. This species’ long-distance acoustic communication, wide geographic distribution, occupation of diverse habitats, and diversity of male acoustic selectivity along its geographic distribution prompted us to evaluate the propagation patterns of calls of different localities and to determine whether or not these patterns provide evidence for the acoustic adaptation hypothesis. To achieve this we: 1) performed a systematic characterization of the geographic variation of advertisement calls emitted by *P. thaul* males; 2) carried out call propagation experiments to assess the propagation properties and degradation of advertisement calls in native and in foreign localities. As such, we tested the prediction of the acoustic adaptation hypothesis that local calls are better transmitted and less degraded than foreign calls. By studying signal propagation patterns in the extremely variable environments inhabited by *P. thaul* we expected to contribute significant knowledge on the evolutionary adaptations of acoustic communication systems.

## Results

### Advertisement calls

**Figure 1:**
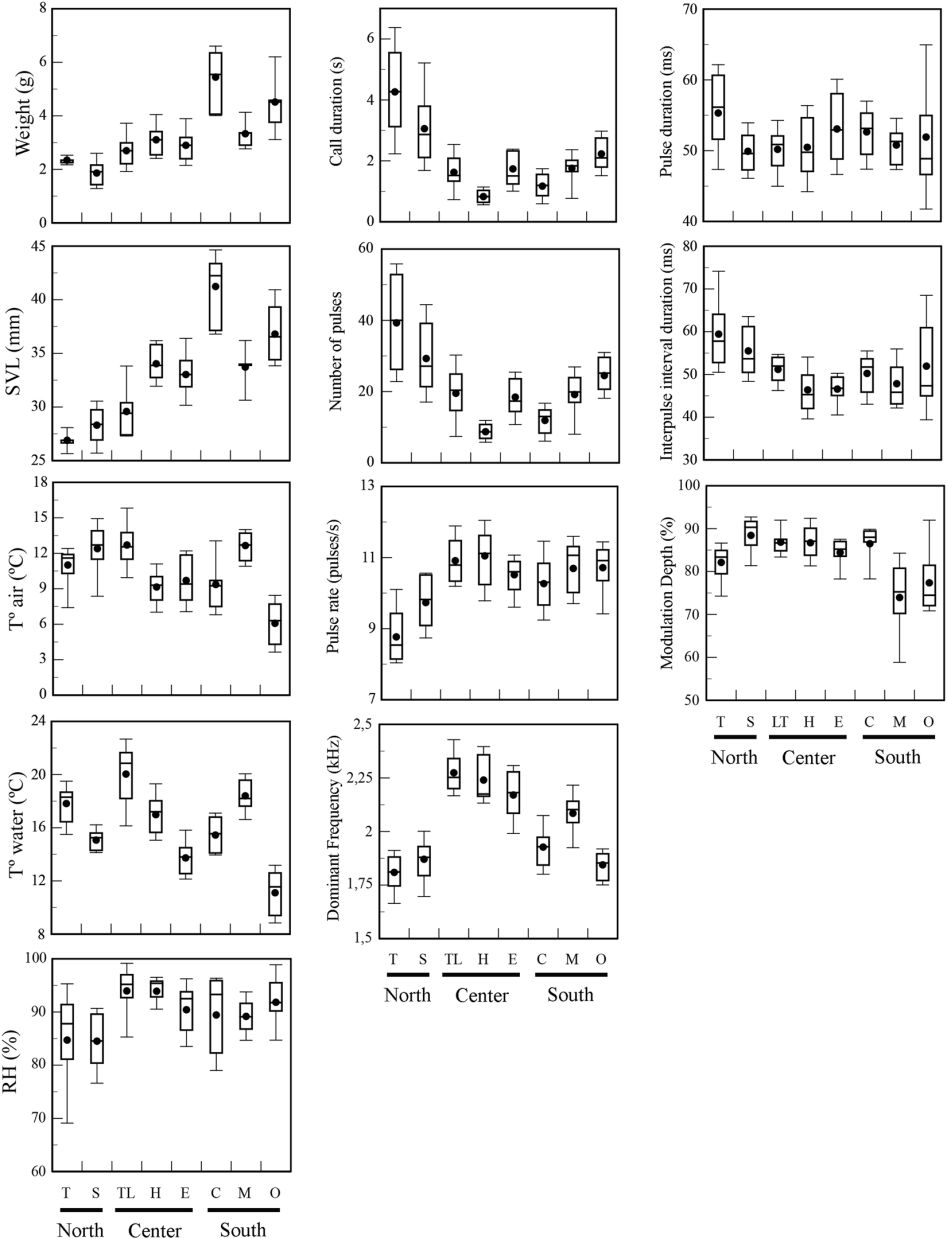
Box plot showing variation in all of the parameters measured in the eight localities studied. The first column shows variation in environmental and *P. thaul* morphological parameters. The second and third columns show variation in acoustic variables of the advertisement calls. T, Totoral; S, Socos; TL, Torca Lagoon, H, Hualqui; E, Elicura, C, Coñaripe; M, Máfil; O, Osorno.

**Figure 2:**
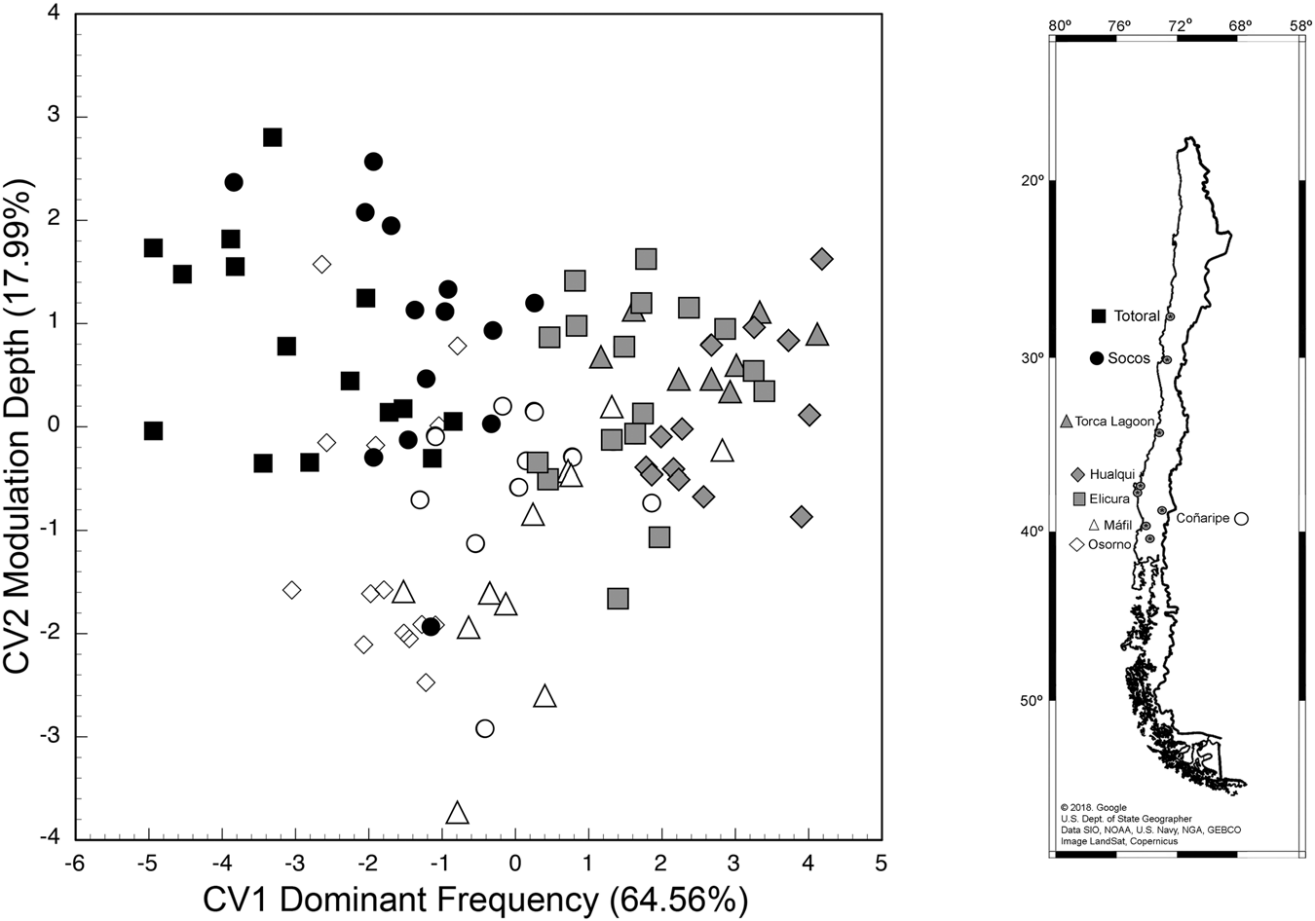
Stepwise discriminant analysis of the advertisement call acoustic parameters for the eight localities where *P. thaul* was studied. Both canonical variables explained nearly 90% of the total variation. CV1 was associated with Dominant frequency and CV2 was associated with Modulation depth. The symbols identifying individuals from different localities are shown in the schematic map on the right. In the bi-plot, black: north, gray: central, and white: south. The map was generated from digital information available at Google Earth Pro v7.3.0.3832 and modified with Adobe Photoshop CS6 v.13.0.6.

Advertisement calls varied considerably among the localities studied along the distribution of *Pleurodema thaul* in Chile. From this variation, it was possible to discriminate three stocks with distinct characteristics, namely northern, central, and southern groups (Stepwise Discriminant Analysis: Wilks’ λ = 0.0261, *F*_49,451_ = 9.6747, *P* < 0.0001; Figs 1 and 2). The first two canonical variables explained approximately 90% of the total variation (CV1: 64.5607% and CV2: 17.9993%). From the correlation between the original acoustic variables and the scores of the canonical variables, we determined that CV1 was significantly associated with dominant frequency (Pearson correlation: r = 0.7519, P < 0.05) and CV2 with modulation depth (Pearson correlation: r = 0.5579, P < 0.05). Thus, these two acoustic variables mostly contributed to call variation between localities. Furthermore, this analysis showed that 67.6% of all individuals were correctly classified within their locality. This value only decreased to 62.7% after applying Jackknife sub-sampling.

All of the environmental and morphological variables measured at the end of the recording session differed significantly among localities (Univariate GLM: SVL: F_7,94_ = 54.588, P < 0.0001; Weigth: F_7,94_ = 32.333, P < 0.0001; Air Temperature (Tair): F_7,94_ = 17,437, P < 0.0001; Water Temperature (Twater): F_7,94_ = 41.261, P < 0.0001; Relative Humidity (RH): F_7,94_ = 4.7211, P < 0.001; Fig. 1). Individuals from southern localities had larger Snout-Vent Lengths (SVLs) and weight as compared to individuals from the northern and central localities. RH was also higher in the southern versus the northern localities. In addition, air temperature was higher in northern than in localities, while no clear pattern in water temperature was found among localities. The central localities had intermediate values between northern and southern localities to both air temperature and RH.

### Propagation experiments

**Figure 3:**
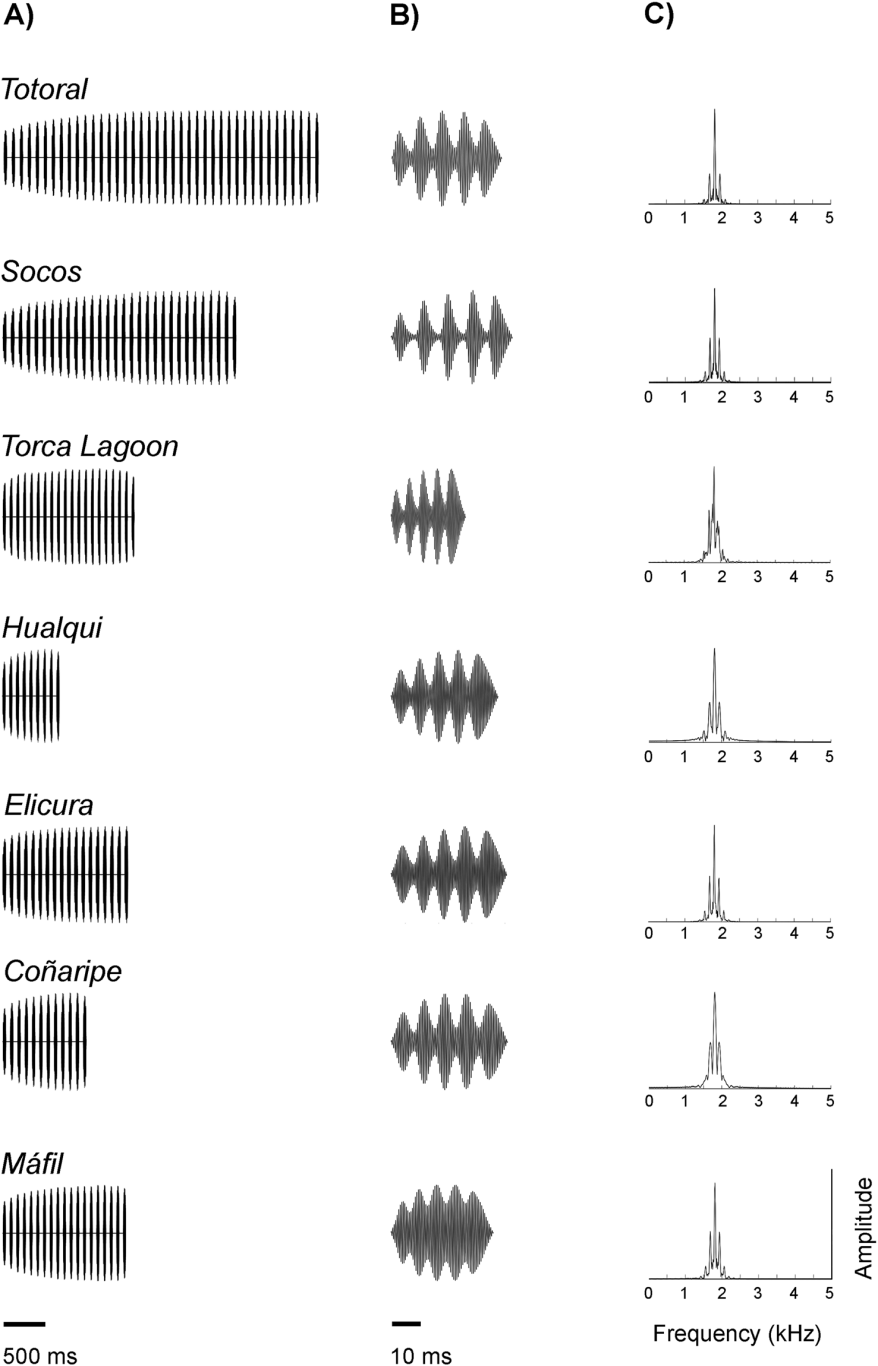
Synthetic advertisement calls generated for seven localities where *P. thaul* was recorded. These were used in propagation experiments. (**A**) Oscillograms of the entire calls, (**B**) expanded view of one pulse of synthetic calls, and (**C**) power spectra of pulses.

**Figure 4:**
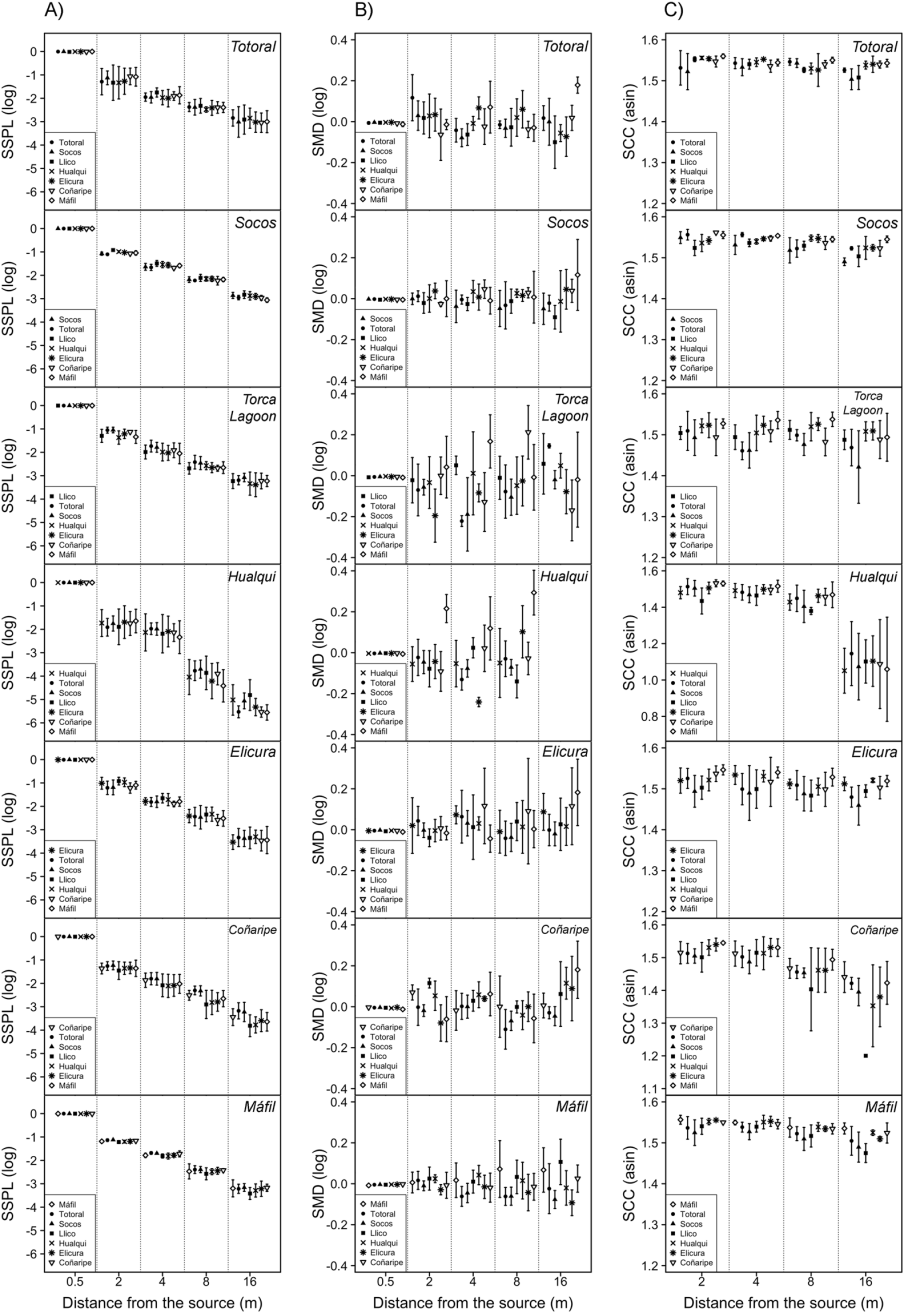
(**A**) Standardized sound pressure level (SSPL), (**B**) standardized modulation depth (SMD) and (**C**) spectral cross-correlation (SCC) for 16-m transects taken in seven *P. thaul* sampling localities.

The influence of environment on the transmission of *P. thaul* advertisement calls was tested by means of propagation experiments. The synthetic advertisement calls used in the propagation experiments are shown in Fig. 3. Propagation distance had a great influence on the propagation of these stimuli; in all localities where we carried out these experiments, the distance from the sound source had a significant effect on call standardized sound pressure level (SSPL). Call origin, namely the different synthetic calls with the characteristics of each locality, had a significant effect on SSPL in most of the localities with the exception of the tests carried out in Totoral and Hualqui. However, no significant interactions between distance and call origin were found at any locality (Table 1, Fig. 4A). The pairwise comparisons between call origins that differed significantly showed that local advertisement calls had better performances than the foreign advertisement calls in a similar number of cases (seven and six comparisons, respectively) (Table 1 of Supplementary material and Fig. 4A).

**Table 1:** Effects of distance from the sound source and call origin on the Standardized Sound Pressure Level of synthetic calls in seven localities where *Pleurodema thaul* was sampled.

	Effect	Numerator df	Denominator df	F	P
Totoral	Distance	4	102	268.4036	0.0000
	Call origin	6	102	0.1189	0.9939
	Interaction	24	102	0.1960	1.0000
Socos	Distance	4	102	5192.6060	0.0000
	Call origin	6	102	5.8239	0.0000
	Interaction	24	102	1.0193	0.4500
Torca Lagoon	Distance	4	102	1367.7317	0.0000
	Call origin	6	102	4.2108	0.0008
	Interaction	24	102	0.6581	0.8800
Hualqui	Distance	4	102	873.4237	0.0000
	Call origin	6	102	1.3346	2.4870
	Interaction	24	102	1.0137	0.4567
Elicura	Distance	4	102	1372.0915	0.0000
	Call origin	6	102	2.2960	0.0404
	Interaction	24	102	0.4668	0.9827
Coñaripe	Distance	4	102	1059.5590	0.0000
	Call origin	6	102	8.1722	0.0000
	Interaction	24	102	1.1128	0.3441
Máfil	Distance	4	102	2367.1641	0.0000
	Call origin	6	102	2.2641	0.0431
	Interaction	24	102	0.3274	0.9987

In contrast with the SSPL results, distance from the sound source did not affect temporal degradation (i.e. standardized modulation depth, SMD) in most of the localities with the exception of Coñaripe. However, SMD was affected by call origin in almost all localities with the exception of Elicura. The interaction between distance and call origin was significant in Totoral, Torca Lagoon, Hualqui, and Coñaripe (Table 2 and Fig. 4B). The significant pairwise comparisons between call origins showed that local advertisement calls had lower temporal degradation in 11 out of 18 comparisons. Specifically in the northern localities, local calls degraded more than foreign calls (five out of six comparisons). In the southern localities, local calls degraded less than foreign calls (five out of five comparisons). Lastly, the central localities yielded intermediate results although these were more similar to the results obtained for the southern localities since in five out of seven comparisons local calls degraded less than foreign calls (Table 2 of Supplementary material and Fig. 4B).

**Table 2:** Effects of distance from the sound source and call origin on the Standardized Modulation Depth of synthetic calls in seven localities where *Pleurodema thaul* was sampled.

	Effect	Numerator df	Denominator df	F	P
Totoral	Distance	4	99.1498	0.9358	0.4465
	Call origin	6	99.2520	2.6126	0.0215
	Interaction	24	99.1068	2.3695	0.0016
Socos	Distance	4	102	0.0501	0.9952
	Call origin	6	102	2.3387	0.0371
	Interaction	24	102	1.0089	0.4626
Torca Lagoon	Distance	4	97.1136	1.3193	0.2682
	Call origin	6	97.1970	2.9186	0.0116
	Interaction	24	97.1309	2.7300	0.0003
Hualqui	Distance	3	76.2993	2.6801	0.0528
	Call origin	6	76.2299	12.5087	0.0000
	Interaction	18	76.1378	4.2066	0.0000
Elicura	Distance	4	102	2.2830	0.0655
	Call origin	6	102	1.1564	0.3356
	Interaction	24	102	0.8717	0.6381
Coñaripe	Distance	4	94.7739	5.1115	0.0009
	Call origin	6	94.4260	2.5742	0.0236
	Interaction	24	94.2329	1.6714	0.0425
Máfil	Distance	4	102	0.2028	0.9363
	Call origin	6	102	4.6479	0.0003
	Interaction	24	102	1.1722	0.2853

In all localities, distance from the sound source had significant effects on spectral degradation measured as the spectral cross-correlation (SCC) between calls measured at 0.5 m and calls measured at farther distances (2, 4, 8, and 16 m) from the sound source. Call origin significantly decreased spectral degradation in all localities except in Hualqui. The interaction between distance and call origin was significant only in Coñaripe (Table 3 and Fig. 4C). Similar to the SMD results, local calls in the northern localities degraded more than foreign calls (six out of seven comparisons). In the southern localities, local calls degraded less than foreign calls (five out of five comparisons). Lastly, the central localities yielded intermediate results although these were more similar to the results obtained for the southern localities since in four out of five comparisons local calls degraded less than foreign calls (Table 3 of Supplementary material and Fig. 4C).

**Table 3:** Effects of distance from the sound source and call origin on the Standardized Cross-Correlation of synthetic calls in seven localities where *Pleurodema thaul* was sampled.

	Effect	Numerator df	Denominator df	F	P
Totoral	Distance	3	54	3.8860	0.0138
	Call origin	6	54	2.4005	0.0396
	Interaction	18	54	1.1252	0.3552
Socos	Distance	3	79.1822	18.1421	0.0000
	Call origin	6	79.1646	6.8514	0.0000
	Interaction	18	79.1129	1.7228	0.0523
Torca Lagoon	Distance	3	81	4.7619	0.0042
	Call origin	6	81	8.0616	0.0000
	Interaction	18	81	0.8345	0.6554
Hualqui	Distance	3	81	123.0873	0.0000
	Call origin	6	81	0.8042	0.5696
	Interaction	18	81	0.2856	0.9979
Elicura	Distance	3	81	4.6564	0.0047
	Call origin	6	81	7.1006	0.0000
	Interaction	18	81	0.5770	0.9062
Coñaripe	Distance	3	78.0118	87.5378	0.0000
	Call origin	6	78.0095	7.6997	0.0000
	Interaction	18	78.0053	2.4367	0.0037
Máfil	Distance	4	81	27.8567	0.0000
	Call origin	6	81	8.8217	0.0000
	Interaction	18	81	0.8256	0.6656

## Discussion

Here we provide evidence of geographic variation in the acoustic parameters of advertisement calls emitted by *Pleurodema thaul* males. Overall, the dominant frequencies of northern localities were similar to those found for southern localities, and central localities had higher values for this spectral measure. In addition, northern and central localities showed similar intra-pulse modulation depths, and southern localities had lower values for this temporal measure. This variation is consistent with studies that have revealed the existence of three bioacoustic groups along the geographical distribution of this species (i.e. northern, central and southern), which is further matched by genetic variation found among these groups^35^. This geographic variation can be accounted for by intrasexual selection processes, since males produce different vocal responses to calls from local versus foreign localities^36^ while females do not have differentiated phonotactic responses^38^. In addition to these results in *P. thaul*, cases of remarkable variation in the acoustic signals have been reported in other anuran species and related to genetic drift^43^, natural selection^14^, or intersexual selection^44^.

Results from the current study support, at least in part, Morton’s hypothesis of acoustic adaptation. Not all calls of all localities propagated best in their native localities. Studies comparing the propagation efficiency of calls of related anurans have reported that the calls of North American cricket frogs^14^ and Iberian midwife toads^15^ inhabiting areas unfavorable for sound propagation are transmitted better in both native and foreign environments than the calls of conspecifics inhabiting sound favorable habitats. The analyses performed in the current study were not aimed at comparing propagation efficiency among localities where the broadcast tests were carried out and therefore it is not possible to rank the study sites according to their properties for sound transmission. However, it could be naively assumed that transmission in the calling sites in southern Chile is less efficient because of the high vegetation coverage in the austral forest region. Should this be the case, our results bear similarity to the studies in the North American and Iberian species since the calls of *P. thaul* from the southern localities were less degraded in both southern and northern localities relative to those from the northern localities. Further studies utilizing pure tone emissions in the different localities are necessary to compare the efficiency of different environments for sound propagation and degradation^11,15^.

As shown in a previous study, the vocal responses of males from the three biogeographic zones to northern and central calls are stronger than responses to calls from southern localities^36^. Furthermore, that study showed that the main components governing preferences for evoked vocal responses are modulation depth and pulse rate. These previous results analyzed together with findings from the current study, showing lower spectral and temporal degradation of calls from southern compared to northern localities, indicate that males producing calls that experience larger degradation give stronger responses to native than to foreign calls. In contrast with male vocal responses, females from different localities along the geographical distribution of this species give similar phonotactic responses to calls from all localities^38^, indicating that female responsiveness is not related to signal susceptibility to degradation. The positive relationship between signal susceptibility to degradation and male responsiveness to acoustic stimuli provides additional support to the tenet that intrasexual selection has been a significant factor shaping the evolution of acoustic communication in *P. thaul*^36^. Furthermore, results of the current study indicate that in absence of an optimum relationship between signal structure and environmental characteristics for sound propagation of the kind proposed by Morton^7^, male response selectivity for acoustic signals may counteract the effects of disadvantageous signal structure. In addition to the high auditory selectivity for synthetic stimuli with the temporal characteristics of conspecific calls^45^, the vocal responses of *P. thaul* males from the central region have low thresholds that are close to auditory thresholds^42^, which indicates high sensitivity for the properties of their calls. Such behavioral sensitivity contrasts with that of other anurans in which thresholds for evoked vocal responses are well above sensory thresholds^46,47^. Both response patterns combined may provide effective mechanisms to counteract the effects of signal degradation and lack of optimality in relationships between signals and natural environments in which these sounds propagate. Studies on the recognition of degraded signal patterns by callers would help to comprehensively understand the effectiveness of sound communication in *P. thaul*. Contrasting capabilities of discrimination of call degradation patterns have been recently reported in diverse species of anurans^48–50^.

The lack of strict optimality between acoustic signal structure and the physical characteristics of the environments where anurans inhabit and call has been related to the dependence of breeding activity on water sources. This dependence restricts the distances over which these vertebrates typically communicate^51^. In addition, microhabitat structure rather than gross environmental landscape features is likely to have more significance for the propagation of anuran signals^31^. Behavioral responses, like the use of resonant calling and listening posts that compensate for signal attenuation and degradation^52^ may contribute additional mechanisms to overcome communicative constraints imposed by environmental conditions.

Given that the number of species calling syntopically at night time in desert and temperate environments is rather low compared to that observed in tropical regions, *P. thaul* calls are subjected to a low diversity of biotic interference^53,54^. In the northern zone, no other anuran species calls at all while in the central zone the only species calling syntopically is *Calyptocephalella gayi*. In contrast with this scarcity, *P. thaul* occurs in sympatry with *Calyptocephalella gayi, Hylorina sylvatica, Eupsophus calcaratus, Eupsophus emiliopugini and Batrachyla antartandica* in the southern zone^11,55^. Further studies are necessary to assess the relevance of soundscapes on the divergence of the acoustic signals of this species along its geographical distribution.

Measurements of thresholds of male vocal responses have shown that *P. thaul* males are highly sensitive to conspecific calls. Males respond at amplitude levels of about 43 dB SPL, matching the midbrain auditory thresholds of this species. These behavioral and neural data combined indicate that this frog communicates over 70 m, a long distance for anurans^42^. Further studies examining the auditory sensitivity of males and females in different localities along the geographic range of *P. thaul* are necessary to establish how sensory capabilities may counteract the attenuation and degradation of propagated calls in natural conditions.

## Methods

### Advertisement calls

We recorded natural advertisement calls at eight localities where *Pleurodema thaul* is known to breed. This effort represents the most comprehensive sampling of the broad distribution range of this species yet undertaken in a study. The localities, dates when the recordings were carried out, and number of recorded individuals included the following: Totoral (27°54′S, 70° 56′W, September 2012, N = 16), Socos (30°43′S, 71° 29′W, October 2012, N = 14), Torca Lagoon (34°46′S, 72°2′W, January 2013, N = 8), Hualqui (36°55′S, 72°51′W, November 2012, N = 13), Elicura (37°55′S, 73°6′W, October 2013, N = 17), Coñaripe (39°32′S, 71°54′W, December 2012, N = 10), Máfil (39°41′S, 72°57′W, November 2012, N = 11), and Osorno (40°35′S, 73°3′W, October 2011 and September 2012, N = 14). For each recorded individual, we analyzed on average 26.6 calls (range: 7–70 calls). According to the geographic distribution of *P. thaul*, we considered the Totoral and Socos localities as belonging to the northern bioacoustic group, Torca Lagoon, Hualqui and Elicura as belonging to the central group, and Coñaripe, Máfil and Osorno as part of the southern group. At each locality we choose sites where interference from anthropogenic noise was negligible. At nighttime (i.e. 20:00-04:00 hrs.), we located frogs calling in slow flowing creeks, pools, and lagoons. Advertisement calls were recorded with an unidirectional microphone (Sennheiser ME66, Sennheiser Electronic GmbH and Co., KG, Wedemark, Germany) placed 20 cm from the focal individual and connected to a digital recorder (Tascam DR-100, TEAC Corporation, Tokyo, Japan). The sampling rate and resolution of the WAV files acquired with the digital recorder were 44.1 kHz and 16 bits, respectively. At the end of a recording session, most of the recorded frogs were captured to measure their body weight (Pocket Pro Balance; Acculab, Precision Weighing Balances, Bradford, MA, USA; ±0.01 g) and Snout-Vent Length (SVL, Digital Caliper; Traceable, Galvestone, TX, USA; ±0.001 mm). Air and water temperature (EXTECH 421502, Extech Instruments Corporation, Waltham, MA, USA) and relative humidity (EXTECH RH390, Extech Instruments Corporation, Waltham, MA, USA) were measured at the end of each recording session. In addition, the latitude and longitude of each locality were recorded using the Global Positioning System (WGS84, Garmin eTrex Vista® H, Garmin International, Inc., Olathe, Kansas, USA). All procedures employed in the present study were previously approved by the Ethics Committee of the University of Chile (CBA 0423 FMUCH) and complied with regulations for animal care and conservation in Chile (Livestock and Agriculture Service permit number 7311).

The sound files were analyzed with a custom-built routine in MATLAB, version 7.5 (MathWorks, Natick, MA, USA). Briefly, for all calls contained in the recorded files, we computed the signal envelope as the modulus of the analytical signal obtained from the Hilbert transform of the time series. Subsequently, we applied an amplitude threshold on this envelope, and a presence (1) or absence (0) logical vector was automatically implemented. When necessary, errors were manually corrected. From this logical vector, all calls were segmented and initial and final times of each call pulse were obtained to calculate the following five temporal variables: call duration, number of pulses, average pulse duration, average inter-pulse interval duration, and pulse rate. In addition, the amplitude modulation depth was calculated as a percentage that corresponded to the ratio of the difference between the maximum and minimum envelope amplitude and the maximum envelope amplitude within a pulse. Finally, the dominant frequency was obtained from the peak of the fast Fourier transform computed for the central pulse of each call^35^. This routine allowed us to perform an automated analysis of the complete recordings of each individual. These temporal and spectral parameters have been used to effectively differentiate calls between localities within this species’ distributional range^35^.

Because call parameters are affected by both environmental and morphological parameters^51^, we calculated correlations between water temperature and temporal variables and between SVL and dominant frequency. The acoustic parameters that were significantly correlated with environmental and morphological variables were corrected as in previous studies^35,56^. As such, we calculated the regression coefficients between acoustic variables and water temperature or SVL to adjust them to the total means of water temperature and SVL. To perform these corrections we used the equation (1):

1 $$Y{\rm{corr}}=Y-(b\ast X{\rm{measured}})+(b\ast X{\rm{mean}})$$

where *Y*_corr_ is the corrected call variable, *Y* is the original value of an acoustic variable, *b* is the regression coefficient, *X*_measured_ is the water temperature or SVL measured after each recording, and *X*_mean_ is the mean value of the acoustic variable to be corrected for all localities. All subsequent analyses were performed with values corrected following this procedure. Univariate GLMs (General Linear models) were carried out to study the variation of each environmental and morphological variable measured. Tukey’s tests were performed as a posteriori comparisons. In addition, stepwise discriminant analyses were used to explore the variation in the acoustic variables and to determine which original acoustic variables best explained the variation among localities. In addition, Jackknife sub-sampling was applied to the classification carried out by the discriminant analysis. All of these analyses were carried out in STATISTICA 8.0 (StatSoft, Inc. Tulsa, Oklahoma, USA).

### Propagation and degradation experiments

To study the transmission and degradation of the advertisement calls of *P. thaul*, we carried out experiments with synthetic advertisement calls generated specifically for each locality. These were evaluated in seven of the original eight localities. The locality of Osorno was excluded from these experiments because the topographical characteristics of this site changed from the time when natural advertisement calls were recorded to the time when propagation experiments were planned to be performed (the land was ploughed and the pool was drained). These experiments were carried out in the rest of the localities during the reproductive seasons of 2013-2014. Using Adobe Audition 3.0 (Adobe System Inc. San José, CA, USA) we built synthetic calls for each locality using a sampling rate of 44.1 kHz and 16 bits of resolution. The synthetic calls were generated using the average values of the acoustic parameters from the analysis carried out on the natural calls recorded at each locality. Because in most localities the advertisement calls had five intra-pulse modulations, we built synthetic advertisement calls containing this feature for all localities. For each locality, five replicates of each stimulus were broadcast with an inter-stimulus interval of 6 s. These stimuli were played back with a third generation Ipod nano (Apple Inc. Cupertino, CA, USA) and using a two-channel impedance-matched operational amplifier and an attenuator (Hewlett**–**Packard 355 C/D, Hewlett**–**Packard Enterprise, Palo Alto, CA, USA). The output signal was amplified (Alpine 3540) and broadcast via a loudspeaker (Polk Audio, MM 10a, Polk Audio, Baltimore, MD, USA). We set up 16-m length transects at each locality where the natural advertisement calls were recorded. In these sites, the loudspeaker was positioned where *P. thaul* males were known to typically call, and five omnidirectional microphones (Sennheiser mkeII, Electronic GmbH & Co., KG, Wedemark, Germany) were placed 0.5, 2, 4, 8 and 16 m from the loudspeaker. Each microphone was connected to a different channel of a digital recorder (Tascam DR-680, TEAC Corporation, Tokyo, Japan) set up to generate WAV files of 44.1 kHz and 16 bit. At the end of each experimental session, the recordings of the synthetic advertisement calls obtained with each of the five microphones were calibrated with a 1-kHz and 94 dB SPL RMS tone delivered with a sound calibrator (Brüel & Kjaer 4231, Brüel & Kjaer Instruments, Inc., Boston, MA, USA) using the same channels and gains as for the propagation experiments. Within each locality the topography of the pools were not always appropriate to set 16-m transects, therefore we were able to conduct only five complete transects in each locality.

To analyze the files generated from propagation experiments, selections of segments containing recorded synthetic advertisement calls were obtained with Raven Pro 1.4 (Cornell Lab of Ornithology, Bioacoustics Research Program, Ithaca, NY, USA). We used these selections to carry out the acoustic analyses. For this, a custom-automated routine was generated in R version 3.2.1 (R Core Team, 2015) using the libraries tuneR^57^ and Seewave^58^. To correctly calculate the SPL values of the calls embedded in noise, we selected segments of background noise contiguous to the recorded calls using Raven Pro 1.4 (Cornell Lab of Ornithology, Bioacoustics Research Program, Ithaca, NY, USA)^59,60^. To obtain the envelope of signals, we performed a Hilbert transform using a smoothing of 400 points and a 0% overlap between windows. From this envelope we calculated the modulation depth as the percentage between the maximum and minimum envelope amplitude values within a pulse of each call. To obtain spectral cross-correlation values (SCC), mean power spectra were obtained with a fast Fourier transform having a window length of 440 points (Window type = Hanning; Overlap = 0%; Frequency resolution = 100.227 Hz; Temporal resolution = 9.977 ms). We obtained the SCC values at zero-lag between the synthetic advertisement calls recorded at the shortest (0.5 m) and the farther distances (i.e. 2, 4, 8 and 16 m) from the loudspeaker. Because environmental noise can interfere with measurements, we visually inspected plots of the envelope and spectra to determine if they were obtained properly, otherwise the segments were discarded from further analyses.

We performed separate analyses for each locality and in order to obtain comparable measurements for each call origin (i.e. locality), individual values of SPL and modulation depth were standardized by dividing individual values by the highest value of the corresponding call origin obtained at the shortest distance (0.5 m). The variables obtained are referred to as standardized sound pressure level (SSPL) and standardized modulation depth (SMD) and were calculated for each transect. SCC was not standardized as these values always referred to the shortest distance (0.5 m). These acoustic parameters obtained from the five replicates per call origin were averaged and then subjected to statistical analyses. Data were analyzed using Linear Mixed-Effects Models. Propagation transects were included as random intercepts in order to account for data dependence. Distance, call origin, and their interactions were included as fixed effects, and the models obtained were fitted using Restricted Maximum Likelihood. The significance of fixed effects was evaluated with type-III *F*-tests using the Kenward-Roger approximation to obtain the corresponding degrees of freedom. We evaluated if excluding data yielding residuals above or below 2.5 standard deviations from the mean (i.e. outliers) influenced the significance of the fixed effects. If results were not influenced by the inclusion of outliers, these data were retained in the analysis. Because our main interest was to evaluate if local calls experience less attenuation and temporal/spectral degradation than all foreign calls, *a priori* contrasts were obtained comparing local calls with all foreign calls when the effect of call origin was significant. If either the distance or the interaction between distance and call origin were significant, additional models were fitted for each distance separately and the same *a priori* contrasts were performed (i.e. local vs. foreign calls). This allowed us to maintain the focus of the analysis on local calls. To improve data normality and model fitting, SSPL and SMD data were log or square-root transformed, and SCC data were arcsine transformed when necessary. These analyses were performed using R 3.2.1 (R Core Team, 2015). Models were fitted using the library lme4^61^; *p*-values were obtained with the library lmerTest^62^, and residuals were evaluated using the library LMERConvenienceFunctions^63^.

## Electronic supplementary material

Supplementary Information
