# Supplementary material for: The acoustic adaptation hypothesis in a widely distributed South American frog: Southernmost signals propagate better
Source: Sci Rep. 2018 May 3;8:6990. doi: 10.1038/s41598-018-25359-y (PMC5934381; doi:10.1038/s41598-018-25359-y)
Supplement: Supplementary file 1 — Supplementary Information [file 41598_2018_25359_MOESM1_ESM.docx]

**The acoustic adaptation hypothesis in a widely distributed South American frog: Southernmost signals propagate better.**

**Nelson A. Velásquez*^1,3^, Felipe N. Moreno-Gómez^2,3^, Enzo Brunetti^3^ & Mario Penna^3^**

^1^ Laboratorio de Communicación Animal, Facultad de Ciencias Básicas, Universidad Católica del Maule, San Miguel 3605, 3480112, Talca, Chile. ^2^ Laboratorio de Bioacústica y Ecología del Comportamiento Animal, Facultad de Ciencias Básicas, Universidad Católica del Maule, San Miguel 3605, 3480112, Talca, Chile. ^3^ Programa de Fisiología y Biofísica, ICBM, Facultad de Medicina, Universidad de Chile, Independencia 1027, Santiago, Chile. * Correspondence and request for materials should be addressed to N.A.V. (email: [nelson.velasquez@gmail.com](mailto:nelson.velasquez@gmail.com)).

**Table 1.** Pairwise comparisons of the effect of call origin on Standarized Sound Pressure Level between local and foreign localities. Abbreviations: S: Socos, M: Máfil, H: Hualqui, TL: Torca Lagoon, T: Totoral, E: Elicura and C: Coñaripe. < > indicate the relative effects of call origin on SSPL of propagating calls in a given locality and at various distances from the sound source, indicated in the third column.

|  | Call origin | Distance (m) |
| --- | --- | --- |
| Socos | S>M | 16 |
|  | S<H | 2-4 |
|  | S<LT | 2-4 |
| Torca Lagoon | TL<T | 2-4-8 |
|  | TL<S | 2-4 |
| Elicura | E>C | 2 |
|  | E>S | 2 |
|  | E>T | 2 |
| Coñaripe | C>H | 16 |
|  | C>TL | 8-16 |
| Máfil | M>TL | 16 |
|  | M<T | 4 |
|  | M<S | 4 |

**Table 2.** Pairwise comparisons of the effect of call origin on Standardized Modulation Depth between local and foreign localities. Abbreviations: T: Totoral, C: Coñaripe, E: Elicura, M: Máfil, S: Socos and TL: Torca Lagoon. < > indicate the relative temporal degradation of call origin in a given locality and at distances from the sound source, indicated in the third column.

|  | Call origin | Distance (m) |
| --- | --- | --- |
| Totoral | T<C | 2 |
|  | T>E | 4 |
|  | T>M | 4-8 |
| Socos | S>C | 2-4-8-16 |
|  | S>E | 2-4-8-16 |
|  | S>M | 2-4-8-16 |
| Torca Lagoon | TL<E | 2 |
|  | TL<S | 4 |
|  | TL<T | 4 |
|  | TL<C | 16 |
|  | TL>C | 8 |
| Hualqui | H<E | 4 |
|  | H>M | 2-4-8 |
| Coñaripe | C<E | 2 |
|  | C<M | 2 |
| Máfil | M<E |  |
|  | M<S |  |
|  | M<T |  |

**Table 3.** Pairwise comparisons of the effect of call origin on Standardized Cross-Correlation between local and foreign localities. Abbreviations: S: Socos T: Totoral, TL: Torca Lagoon, C: Coñaripe, M: Máfil, E: Elicura and H: Hualqui. < > indicate the relative spectral degradation of call origin in a given locality and at distances from the sound source, indicated in the third column.

|  | Call origin | Distance (m) |
| --- | --- | --- |
| Socos | S<TL | 2 |
|  | S>C | 4 |
|  | S>M | 4 |
|  | S>T | 4 |
|  | S>E | 8-16 |
|  | S>H | 8-16 |
|  | S>M | 8-16 |
| Torca Lagoon | TL<T | 4 |
|  | TL<S | 16 |
|  | TL<M | 4 |
| Elicura | E<S | 4-16 |
|  | E<T | 4-16 |
| Coñaripe | C<S | 4 |
|  | C<H | 16 |
|  | C<TL | 8-16 |
| Máfil | M<S | 2-4-16 |
|  | M<TL | 16 |
